# Unique repertoire of anti-carbohydrate antibodies in individual human serum

**DOI:** 10.1038/s41598-020-71967-y

**Published:** 2020-09-22

**Authors:** Ralph N. D. Luetscher, Tanya R. McKitrick, Chao Gao, Akul Y. Mehta, Alyssa M. McQuillan, Robert Kardish, Kayluz Frias Boligan, Xuezheng Song, Lenette Lu, Jamie Heimburg-Molinaro, Stephan von Gunten, Galit Alter, Richard D. Cummings

**Affiliations:** 1 Department of Surgery, Harvard Medical School, Beth Israel Deaconess Medical Center, National Center for Functional Glycomics, CLS 11087 – 3 Blackfan Circle, Boston, MA 02115 USA; 2 Institute of Pharmacology, University of Bern, 3010 Bern, Switzerland; 3 Department of Biochemistry, Emory University School of Medicine, Atlanta, GA 30303 USA; 4 The Ragon Institute of MGH, MIT, and Harvard, Cambridge, MA 02139 USA; 5 Present Address: Department of Biology, Institute of Microbiology, ETH Zurich, 8093 Zurich, Switzerland; 6 Scienion US, 2640 West Medtronic Way, Tempe, AZ 85281 USA; 7 Present Address: Division of Infectious Diseases and Geographic Medicine, Department of Internal Medicine, UT Southwestern Medical Center, 5323 Harry Hines Blvd, Dallas, TX 75390 USA

**Keywords:** Glycobiology, Antibodies

## Abstract

Humoral immunity to pathogens and other environmental challenges is paramount to maintain normal health, and individuals lacking or unable to make antibodies are at risk. Recent studies indicate that many human protective antibodies are against carbohydrate antigens; however, little is known about repertoires and individual variation of anti-carbohydrate antibodies in healthy individuals. Here we analyzed anti-carbohydrate antibody repertoires (ACARs) of 105 healthy individual adult donors, aged 20–60^+^ from different ethnic backgrounds to explore variations in antibodies, as defined by binding to glycan microarrays and by affinity purification. Using microarrays that contained > 1,000 glycans, including antigens from animal cells and microbes, we profiled the IgG and IgM ACARs from all donors. Each donor expressed many ACAs, but had a relatively unique ACAR, which included unanticipated antibodies to carbohydrate antigens not well studied, such as chitin oligosaccharides, Forssman-related antigens, globo-type antigens, and bacterial glycans. We also saw some expected antibodies to ABO(H) blood group and α-Gal-type antigens, although these also varied among individuals. Analysis suggests differences in ACARs are associated with ethnicity and age. Thus, each individual ACAR is relatively unique, suggesting that individualized information could be useful in precision medicine for predicting and monitoring immune health and resistance to disease.

## Introduction

Much of human immunity to microbes and other pathogens is generated against both protein and carbohydrate antigens, and many new vaccines targeting induction of microbial immunity rely on carbohydrate-based conjugate vaccines^1–5^. Yet, while the antigenicity of carbohydrates has long been appreciated in regard to the ABO(H) antigens and related blood groups, the presence of other types of anti-carbohydrate antibodies (ACAs) has been both provocative and poorly understood. Pure polysaccharides are considered to be relatively weak antigens in mammals when used as immunogens alone or used independently of their expression on a microbe, as such free polysaccharides are often T cell-independent and generate a biased IgM response^6–8^. Some zwitterionic polysaccharides can directly induce T cell-dependent IgG anti-carbohydrate antibodies^9,10^, and ACAs to the gut microbiota have been identified^11,12^. Regardless of whether responses are T cell-independent or -dependent, the presence of a diverse array of IgG antibodies to carbohydrates has been observed in many preparations of IVIG, purified IgG products prepared from pooled human plasma and made available through many different commercial sources^13^. Thus, human serum collectively contains IgG that recognizes glycan antigens, including microbial polysaccharides, ABO(H) blood groups, Lewis structures, P blood groups, and the α-galactosyl antigen^11,13–22^. There is uncertainty as to which isotype the anti-blood group antibodies in each individual are, as well as what the breadth of the repertoire of such ACAs might be; studies suggest that both IgM and IgG to the same carbohydrate antigens are found in some individuals, though they may vary in relative ratios^23,24^.

Recently, we identified individuals with primary antibody deficiencies (PADs) who lack IgG to carbohydrate antigens^25^, associated with their propensity for infections^26^ and inability to develop immunity to many carbohydrate-based vaccines. As to the origin of ACAs in general, there is strong evidence that specific ACAs can be induced by exposure to or by infections with different organisms^11,27,28^. Examples include production of antibodies to GM1 ganglioside associated with Campylobacter infection and subsequent Guillain-Barré Syndrome^29–31^, GD1b (lactone) associated with Lyme disease and *Borrelia burgdorferi* infection^32,33^, Tn antigen associated with *Cryptosporidium parvum* infection^34^, and others^35–44^. Thus, ACAs are biologically important, as they serve as primary defense toward microbial pathogens and are the target response to vaccines and vaccine components^27,45–47^. The glycomes of all microbes, fungi, plants, and animals to which humans are exposed are highly complex and represent many millions of different glycan structures^48,49^.

It might be predicted that highly individualistic differences in ACARs occur, since environmental exposures, including microbial exposure, are likely be unique to each individual. Such information may lead to novel insights into human health and personalized medicine, and perhaps address concerns over the importance of vaccination. However, these types of studies have been difficult to implement, largely due to the lack of robust screening technologies. Recent technological developments have employed glycan microarray technology^11,17,19,50–52^ or other array-type strategies^53,54^ to investigate unique individual ACARs. A few studies have focused on bacterial polysaccharides and blood group antigens^18^, and used either pooled sera or total immunoglobulins to assess the ACA diversity^13,14^ on a population level. These studies, although typically focused on a relatively small number of glycans, nevertheless suggest that individuals may differ in specific ACAs depending on the antigen^55–57^, and that some ACAs may be useful in monitoring disorders or transplant antigens^21,50,58^. Others have suggested that ACAs are only to short fragments of glycans but not to longer chains^55^, thus limiting their potential for autoimmune responses. While some studies have suggested shared antibody specificities between individuals^16^ which could be the result of common microbial exposures, overlapping microbiota compositions, or other factors, others have observed differences^17^. Clearly more information is needed in regard to individual differences in ACARs and their relationship to ethnicity, gender and age, as well as exploring recognition of a wider diversity of glycan antigens.

Here we describe our results examining the ACARs on a diverse set of apparently healthy individual donors differing in age, ethnicity, and gender, using glycan microarrays containing novel antigens, including bacterial glycans. The results indicate that the ACAR of an individual can be thought of as a type of “ACAR-barcode” in which the ACAR readout is relatively personal and unique. Within this unique barcode, we identified multiple carbohydrate antigens to which a majority of donors contained antibodies, many of which were unexpected, and suggests that studying the ‘bars’ with commonalities as well as specific differences between individual ACAR-barcodes is an important factor to understanding immunity. The results open new opportunities to explore the broader human ACAR and to define whether ACARs could be useful in terms of predicting disease, health outcomes, and in personalized medicine.

## Methods

### Serum samples

All serum samples were obtained through the Ragon Institute Healthy Control Cohort Study comprised of healthy 18–65-year-olds in the Boston area. The Massachusetts General Hospital Institutional Review Boards (IRB) approved the study, and each subject provided written informed consent for participation in the study; all experiments were performed in accordance with this protocol following the approved, relevant guidelines and regulations. The 105 samples were obtained from 49 females and 56 males between the ages of 20 and 60 + years. The patients were categorized into five age classes, 20–29 years old, 30–39, 40–49, 50–59, and older than 60 years old; therefore, each class consists of approximately 20 patients. The donors were also classified by their ethnicity: 49 Caucasian (Ca), 47 African-American (AA), and 9 Hispanic (Hi). All samples were stored at − 80 °C until use.

### Microarray and binding assay

Multiple glycan microarrays were used. Serum samples were profiled on the NCFGv1 microarray containing 99 different carbohydrates, all printed in quadruplicates. This smaller array was created to represent a subset of major glycan antigens identified from the over 1,000 glycans screened in this study, as described below. The carbohydrates were conjugated with the bi-functional fluorescent linker AEAB at the reducing end by reductive amination to form glycan-AEABs, and then covalently printed at equivalent concentrations on NHS-coated microarray slides as previously described^59^. All serum samples were diluted 1:50 for detecting IgG or IgM, based on preliminary and previous studies^13,14,34^, in TSM Binding Buffer (TSM buffer, 0.05% Tween20, 1% BSA-protease free) and then 115 µL of the diluted serum sample was added to each microarray and incubated on a shaker at RT for one hour. The arrays were washed 8 × with Wash Buffer (TSM buffer, 0.05% Tween20), then 115 µL of the respective secondary detection reagent was added. The secondary antibodies were diluted in Binding Buffer to a final concentration of 5 μg/mL and incubated on the array for one hour, shaking at RT. Arrays were washed four times with Wash buffer, TSM buffer, and finally MilliQ water, before the slides were dried by centrifugation. The arrays were then ready to be scanned and analyzed (see Scanning and Image analysis). The CFG and MGM arrays were acquired from the Consortium for Functional Glycomics (CFG) and were processed in the CFG core facility according to their standard methodology (www.functionalglycomics.org and www.ncfg.hms.harvard.edu). All of the bound antibodies of the 105 serum samples were detected with Cy3-conjugated AffiniPure Goat anti-Human IgG, Fc_γ_ fragment specific (Jackson ImmunoResearch) and Alexa Fluor 647-conjugated AffiniPure Goat Anti-Human IgM, Fc_5μ_ fragment specific (Jackson ImmunoResearch), which are very specific for each antibody type and do not cross-react with other isotypes.

### Scanning and image analysis

Slides were scanned with a GenePix 4300A microarray scanner (Molecular Devices, LLC) and analyzed with the GenePix Pro 7 software. The scan-resolution was 10 μm; IgG were scanned with laserpower 100% and PMT gain 450 and IgM with laserpower 70% and PMT gain 450. The spots were defined as circular features, whereas the final exact alignment and spot resizing was performed manually. Technical faults, like missing spots or dust contaminations were flagged and later excluded from further analysis. Binding intensity was determined by the Relative Fluorescence Unit (RFU), which was calculated by measuring the background subtracted fluorescent average of four replicate glycan spots. In addition, the standard deviation and coefficient of variance (% CV) were calculated for each glycan.

### ACA pull down

To isolate anti-chitin immunoglobulins, 50 µl of chitin magnetic beads (NEB E8036) were washed with 1 × PBS tween-20 (0.05%) three times and incubated with 200 µl of human serum rotating overnight at 4 °C. The chitin beads were washed four times with 1 ml of 1 × PBS tween-20 (0.05%) and eluted with 50 µl of 50 mM glycine–HCl (pH 2.7) for 30 s. The eluted sample was immediately neutralized with 5 µl of 1 M Tris-Base (pH 8) and run on the NCFGv1 microarray. To isolate immunoglobulins against the isoForssman antigen, 100 µl of a 100 µM solution of AEAB labeled glycan was coupled to 50 µl of Pierce NHS-Activated Magnetic Beads (ThermoFisher Scientific). After coupling, the beads were blocked with 3 M ethanolamine (pH 9.0) for 2 h, rotating at room temperature. Isolation of the anti-isoForssman antibodies was done as described above.

### Statistical analysis

Heatmap and hierarchical clustering were performed using the package gplots by Warnes GR, Bolker B, Bonebakker L, Gentleman R, Huber W, Liaw A, Lumley T, Maechler M, Magnusson A, Moeller S, Schwartz M, Venables B. (2015) Various R Programming Tools for Plotting Data. This uses the R package version 2.17.0., within the “R” environment (The R Foundation for Statistical Computing, Version 3.0.2), available at https://cran.r-project.org/web/packages/gplots/gplots.pdf. Other illustrations were performed using GraphPad PRISM (Graphpad Software, Inc., Version 6.0c). Frequency calculations for each clique were done using Microsoft Excel (Microsoft Corporation, 2011, Version 14.0.0).

## Results

### Human anti-carbohydrate IgG and IgM repertoires on the NCFGv1 glycan microarray

To explore the ACAR of healthy people, we used serum samples from 105 individuals of different ages, genders, and ethnicity (Supplementary Table S1), comprised of 49 females and 56 males who were further identified as 49 Caucasians, 47 African-Americans, and 9 Hispanics. We screened each individual serum sample for the presence of bound IgG and IgM, using secondary reagents that we determined were specific for either IgG or IgM with no detectable cross-reactivity, as described in Materials and Methods.

The serum of each individual was screened on one or more of a variety of glycan microarrays. All samples were screened on the NCFGv1 microarray, a designer array that contains 99 glycans, chosen to represent relatively common determinants, including β- and α-glucans, chitins, lacto and globo sialylated series, gangliosides, Forssman and P antigens, Lewis a/b and x antigen types, α-galactosyl antigens (Galα1-3-R), globo- and isoglobo series, as well as the major blood group antigens ABO(H) (Supplementary Table S2, includes common names and glycan structures). Two other glycan microarrays were also used to screen a subset of the healthy individuals; the 610-glycan microarray (v5.1) of the Consortium for Functional Glycomics (CFG), and the 313 glycans on the Microbial Glycan Microarray (MGM) of the CFG (www.functionalglycomics.org). Thus, overall our study employs three arrays which represent 1,022 glycans, and is the largest and most comprehensive number of glycans available to date to identify and compare the unique specificity of ACAs.

**Figure 1:**
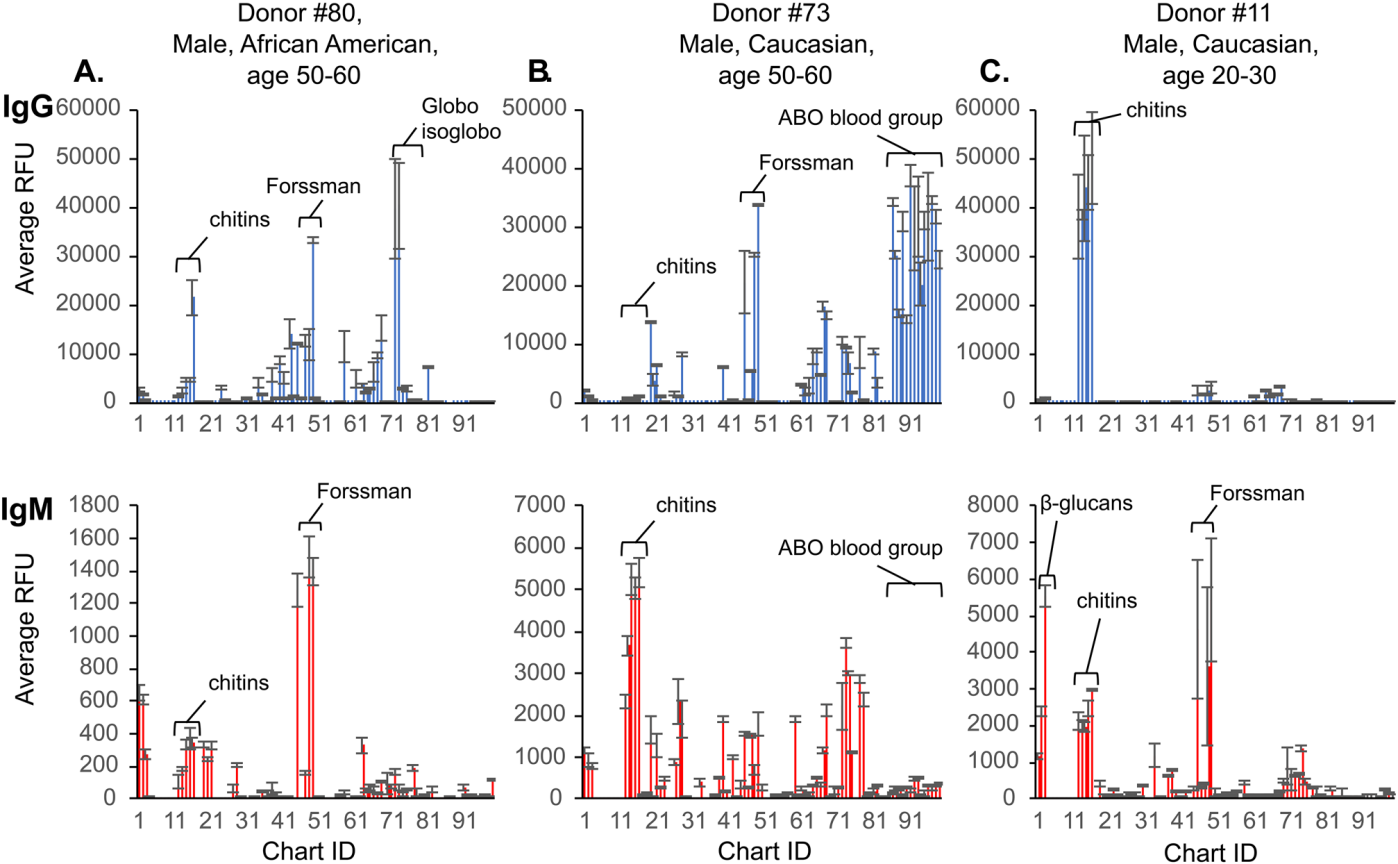
Examples of the anti-carbohydrate antibody repertoires (ACAR) for both IgG (top panels) and IgM (bottom panels) on the NCFGv1 microarray. Data collected from (**A**) Donor #80- an African American male, age 50–59, (**B**) Donor #73- a Caucasian male, age 50–59, **(C)** Donor #11- a Caucasian male, age 20–29. Notable glycan structure categories are named and noted by brackets. RFU = relative fluorescent units. Chart IDs correspond to Supplementary Table S2.

Each of the 105 donor samples differed in their pattern of recognition of glycans. Three examples of the data from individual sera on the NCFGv1 microarray are shown in Fig. 1A–C. Here some of the salient differences noted in a few of the donors are highlighted. For the donor whose data is shown in Fig. 1A, there is both IgG and IgM to chitin tri- to hepta-saccharides, and little antibody reactivity to ABO(H) blood group antigens except for the presence of IgG to #81 Isoglobo-H. This donor also has IgG and IgM antibodies to Forssman antigen pentaose #45, Forssman antigen triose #48, and isoForssman antigen pentaose #49. By contrast, another donor (Fig. 1B) lacks IgG to chitin oligosaccharides, but contains IgM to such antigens; that donor exhibits robust IgG to A and B antigens, but little IgM to those same antigens. While this donor also has antibody to the internal Lewis antigen lacto-N-fucopentaose V (LNFP-V) #59, the above donor (Fig. 1A) lacks antibody to this antigen. Finally, a third donor (Fig. 1C) has very robust IgG to chitin oligosaccharides and some IgM to chitin, but has little IgG to other glycans, and expresses IgM to several oligosaccharides including β-glucan tri- to pentaoses #1, #2, and #3. These three donors exemplify the unique pattern of each individual ACAR that we observed throughout the samples.

**Figure 2:**
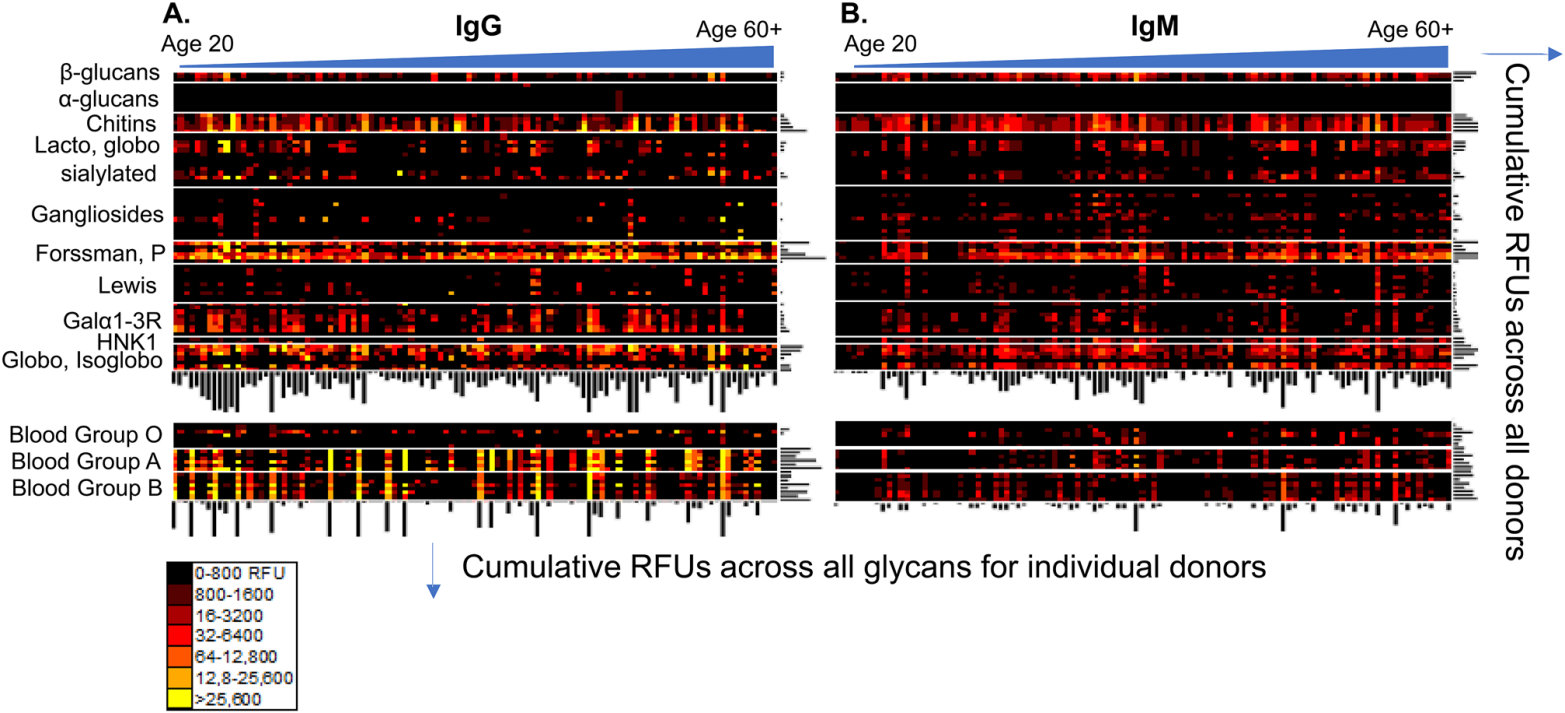
The binding profiles of the entire data set for (**A**) IgG and (**B**) IgM are represented in the form of a heatmap. The individual donor profile is organized vertically, and grouped according to the glycan family listed on the left side. Individuals are ordered left to right, from youngest to oldest. The bar plot on the right side of the heat map represents the cumulative RFUs across all donors, revealing the most predominate anti-glycan antibodies found throughout the sample set. The bar plot on the bottom of the heat map represents the cumulative RFUs within each individual donor. *RFU* relative fluorescent units, *BG* blood group. RFU value ranges and corresponding colors are provided in the key.

The compiled data of the ACAR in the 105 donors is presented as IgG and IgM heatmaps (Fig. 2A, B). The relative fluorescent intensities were binned into a heat map with responses below 800 relative fluorescence units (RFU) marked in black and a signal intensity over 25,600 RFU highlighted as bright yellow, with a range of colors in between to denote the RFU range. This heatmap presents an overall view of the ACAR for the entire sample set and illustrates unique individual patterns (Fig. 2). The donors are classified into one of five different age classes, each class representing a decade, beginning with 20 years old up to donors that are older than 60 years, with each age class containing subclasses for gender and ethnicity. The 105 donors’ individual ACARs are represented vertically in the heat map, and are ordered from left to right as delineated in Supplementary Table S1. The different glycans are ordered by their carbohydrate structure families and the blood group antigens are separately grouped on the bottom of the map. The bar plots on the right side of the heat map represent the cumulative anti-carbohydrate IgG or IgM signal summed for all donors for each glycan; this helps to visualize the highest and lowest bound glycans, such as the Forssman and P antigens. The bar plots on the bottom of the heatmap represent the cumulative IgG or IgM signal intensity summed for all glycans for each donor, denoting the wide range of ACA for each individual. This provides a second, collective manner in which to visualize and explore those glycans that are most intensely recognized by ACA, as well as to view the sum of donors and their most commonly recognized glycans.

### Comparative analysis of all samples for IgG and IgM

**Figure 3:**
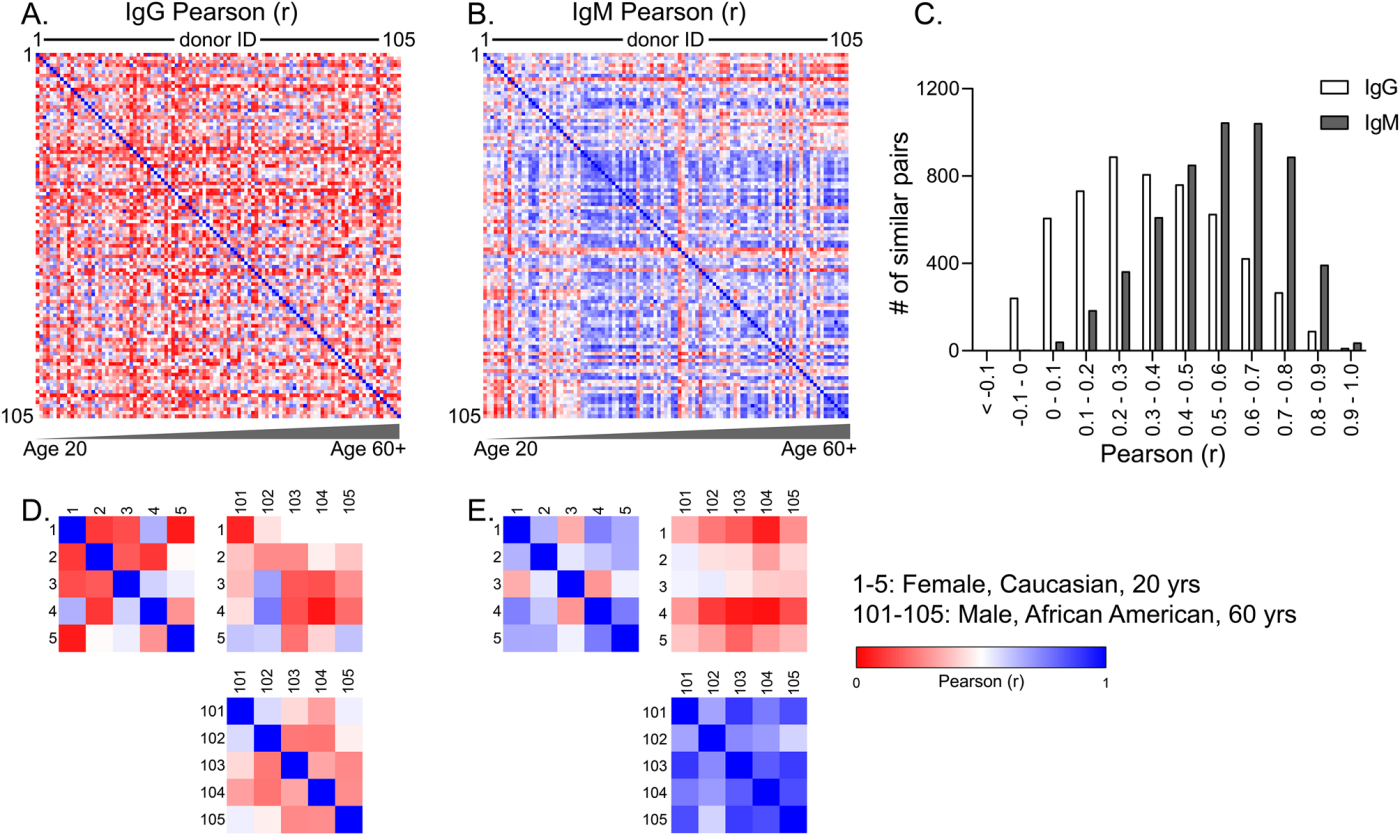
Heatmap of the correlation matrix generated by the Pearson r correlation coefficient for both (**A**) IgG and (**B**) IgM for each individual donor. The scale is set from 0 (red) to 1 (blue), as none of the sample comparisons yielded a negative correlation coefficient. Individuals are plotted in order of donor 1–105 for both heatmaps. (**C**) The distribution and frequency of highly correlated individuals, as shown by Pearson correlation. A subset of the correlation matrix comparing individuals 1–5, with 101–105 for IgG is shown in (**D**) and IgM (**E**).

In order to assess the relationship between the 105 individual samples, we performed a Pearson correlation calculation (*r*)^60^ for IgG and IgM binding data for the NCFGv1 microarray. The Pearson correlation coefficient is a statistically independent method of assessing the patterns of antibody recognition between individuals that is independent of the intensity values (RFU). To calculate the r values between each pair of individuals, the RFUs for each glycan across the array from one individual are compared to those of another individual. The *r* value can range between − 1 (opposite binding trend and negatively correlated) to 0 (no binding trend) to + 1 (similar binding trend and positively correlated). The correlation matrix is presented as a heatmap for both IgG (Fig. 3A) and IgM (Fig. 3B), where the *r* value between each individual is compared against another and plotted in the order from young to old donors. Since no pair of samples showed strong negative correlation (the lowest value was > -0.15), the color scale for the heatmap was set to red = 0, white = 0.5 and blue = 1.

Our results demonstrate that the ACAR of human IgG (Fig. 3A) is less similar between individuals when compared to IgM (Fig. 3B). Regardless of age, gender, and ethnicity, the IgG ACAR appears to be relatively unique, and rarely are two individuals exhibiting even somewhat similar binding profiles. However, this result was not observed in the IgM profiles, which become much more similar with age. The frequency distribution of highly correlated individuals is plotted for both IgG and IgM (Fig. 3C), and clearly demonstrates that the 105 donors have far more similar IgM binding profiles than IgG. A subset of the correlation matrix is shown for IgG (Fig. 3D) and IgM (Fig. 3E), where we are comparing the r values generated by comparing donors 1–5 (Female, Caucasian, 20–29 years) with donors 101–105 (Male, African American, > 60 years). We observed that for the IgM repertoire, older individuals have a much higher correlation coefficient to others of the same age cohort (age > 60 vs. > 60), than to younger donors (age > 60 vs. age 20–29) (Fig. 3E). This suggests that with age, the IgM binding repertoire appears to become more similar, though no such trends are evident from the IgG data (Fig. 3D). However, a few pairs of donors show relatively high correlation in both IgG and IgM binding to the NCFGv1 microarray. For example, Supplementary Table S3 shows IgGs of 6 pairs of individuals with an *r* value of > 0.98 who also show very high correlation with each other for their IgM binding (*r* > 0.9). Nevertheless, in general such cases of extremely high correlation matches are rare, especially for both IgG and IgM. Furthermore, it should be noted that two individuals with an *r* value of > 0.98 for IgG binding on the NCFGv1 microarray, differ significantly in other glycan microarray platforms, thus indicating that overall their ACARs are relatively unique.

**Figure 4:**
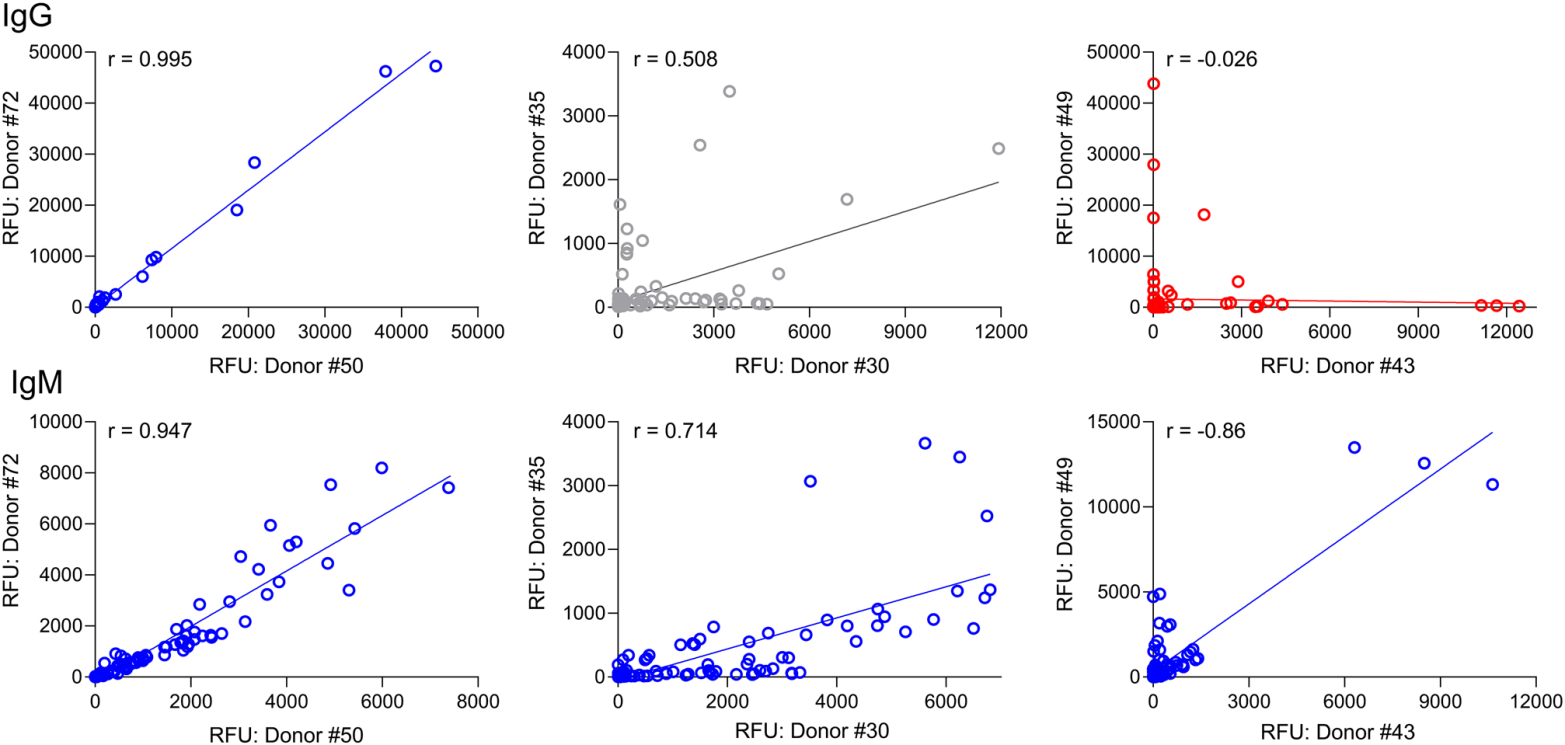
The direct comparison between the signal intensity observed between selected individuals. Each panel compares 2 individuals as notes by donor number. (**A**) IgG correlation, (**B**) IgM correlation. RFU values are represented on the x and y axes, Pearson *r* values are shown above each plot. Samples with a correlation coefficient greater than 0.7 are plotted in blue, between 0.3 and 0.7 are plotted in gray, and less than 0.3 are plotted in red.

As previously stated, there was a range of *r* values observed for the IgG ACAR, from highly correlated to minimal correlation. The question becomes then, if two individuals have a high correlation in their IgG ACAR, does this correspond to a high correlation of their IgM ACAR? In Fig. 4, each graph represents a direct comparison between the signal intensity observed between two individuals, where the points on the plot represent the RFU for every glycan. The data, which are representative of 3 donor pairs with high, moderate, and low correlation between IgG and IgM, reveal that two individuals that exhibit a low correlation between their IgG profiles do not necessarily have a low correlation between their IgM profiles, suggesting that the ACAR is independent of the Ig type.

**Figure 5:**
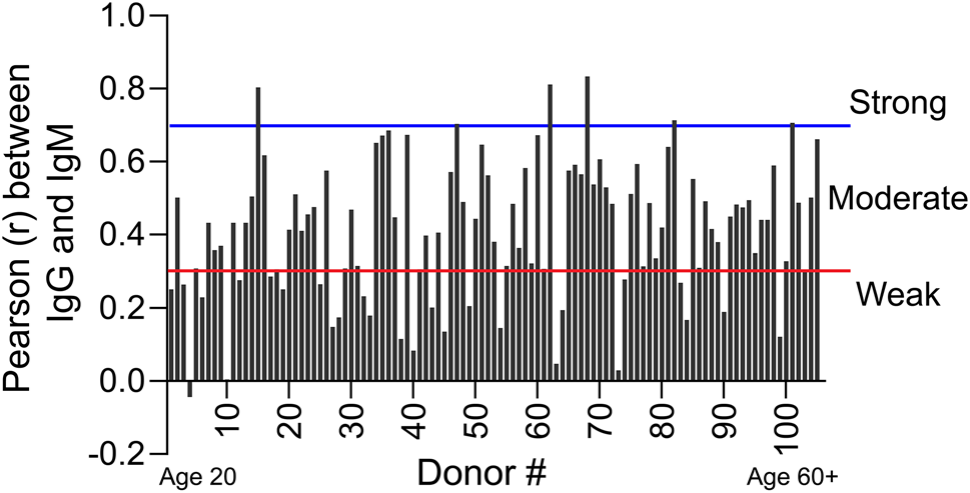
The strength of the correlation between the IgG and IgM profiles within each individual revealed that the majority of IgG and IgM specificities are moderately (between the red and blue lines, n = 72) or weakly (below the red line, n = 27) correlated. Strong correlation- above the blue line (n = 6).

Interestingly, only six individuals in the entire data set had a relatively strong correlation (*r* > 0.7) between their own IgG and IgM binding profiles (Fig. 5). Approximately 25% of the sample set showed no correlation between IgG and IgM (*r* < 0.3) and the remaining ~ 70% showed weak to moderate correlations (0.3 < *r* < 0.7), and these trends were not driven by gender, age or ethnicity. Based on historical presumptions, one might predict that the IgM and IgG profiles would be more highly correlated; however, this trend was not observed in the data. Thus, we conclude that the presence of IgM anti-carbohydrate antibodies is not predictive of IgG antibodies to the same glycan antigens, and vice versa. Interestingly, in this regard, there were a few individuals who expressed little IgG to any of the glycans on the NCFGv1 microarray, but had significant IgM to a variety of glycans (donors 35 and 36), whereas some others had significant IgG, but lacked IgM, to glycans (donors 7 and 8).

### ACARs to different glycan targets on other glycan microarrays

**Figure 6:**
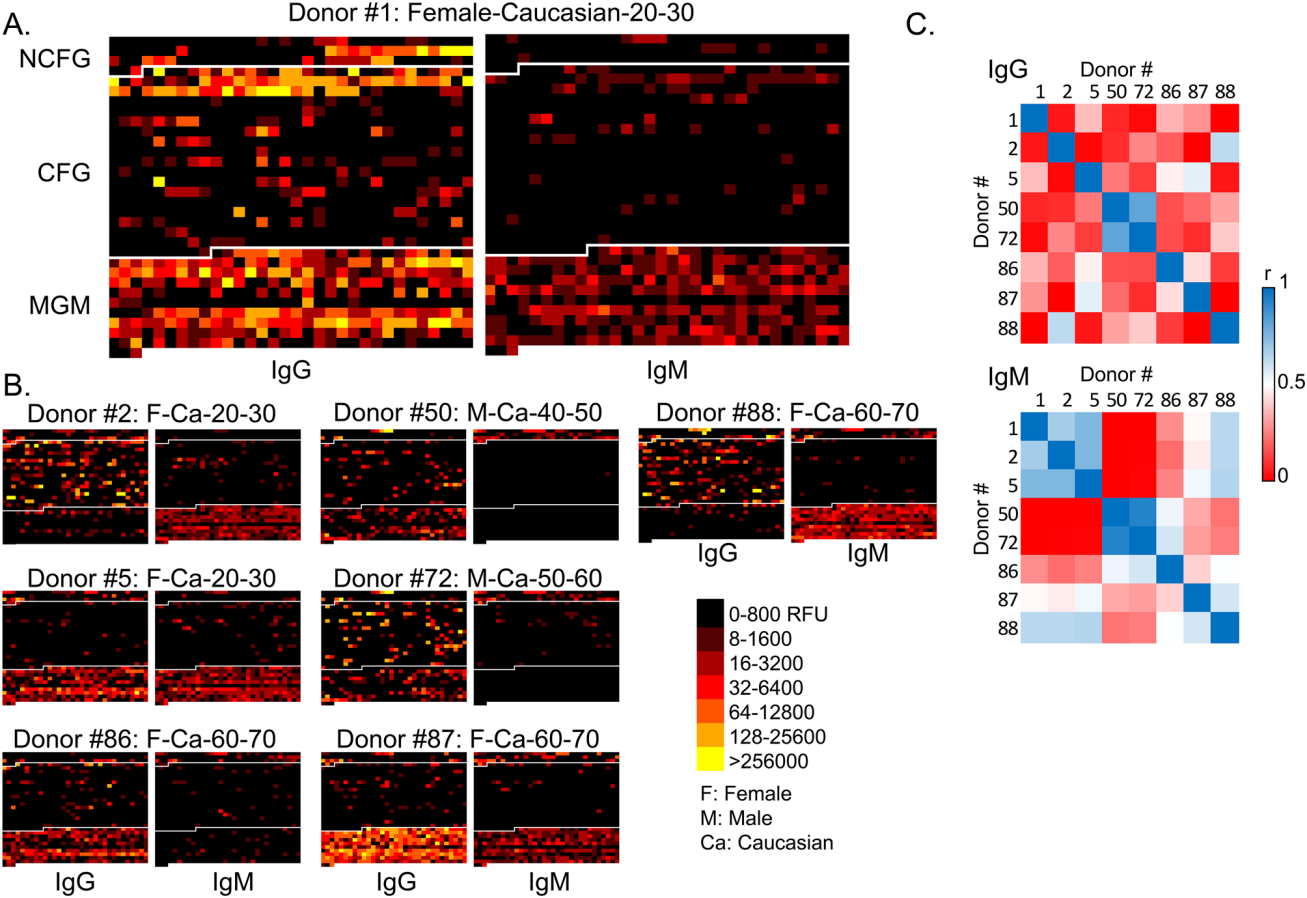
Total ACAR profiles of 8 selected donors over ~ 1,000 glycans, represented in a heatmap of the IgG (left panels) and IgM (right panels) screened on the NCFGv1 microarray (top sections), CFG (middle sections) and MGM (bottom sections) glycan microarrays. Data from each array are separated by a white line. Donor #1 (**A**) is made larger to see the profiles more clearly, followed by the remaining 7 profiles (**B**). (**C**) Pearson correlation coefficient map of the same 8 individuals, IgG (top) and IgM (bottom).

For a subset of individuals (n = 8), we screened the donor serum on the mammalian glycan (CFG) and pathogen arrays (MGM) to determine if the trends we observed could be extended to a different set of target glycans. These data are presented as individual heat maps, with the IgG and IgM binding patterns of ~ 1,000 glycans included in each block. One individual donor (#1) is shown in Fig. 6A in a larger view, and other individuals are shown at smaller scale in Fig. 6B. As observed previously, each individual serum had a relatively unique IgG profile on all three types of glycan arrays and a low level of similarity was seen between individuals (Fig. 6C). The pattern was similar for IgM binding, as well, where individuals within a particular age cohort appeared to be more similar than individuals in different age cohorts. Notably, the lower portions of each block represent binding to the MGM array, which contains bacterial polysaccharide-type antigens. Binding patterns range from barely detectable to very high levels, which are likely reflective of the collection of bacteria that each individual has encountered. This diversity in response of both IgG and IgM indicates that robust responses to bacterial glycan antigens are elicited in humans, but the ACAR for those antigens also differ among all individuals tested. Altogether, the analysis of the 8 individuals on the additional glycan arrays confirmed the conclusions drawn from analyses on the NCFGv1 microarray, namely that the ACAR of each individual is unique among all glycan antigens tested, and the binding data generates a type of ‘ACAR-barcode’ for each individual.

### The human ACAR is unique for an individual but shows some dominant patterns for certain antigenic glycans

While the ACAR is relatively unique for each individual, there were antibodies to some glycans unrelated to ABO(H) blood groups that were unexpectedly present in many donors. Surprisingly, the majority of sera tested have IgG and IgM signals toward chitin oligosaccharides, Forssman, and globo antigens; they were dominant relative to the α-Gal antigens, which have been reported to be dominant in human sera^15,61^. Obviously, such historical publications should be viewed with caution, as more glycans are now available to screen for anti-carbohydrate responses, as shown here. Many individuals had no detectable antibody to certain α-Gal antigens, whereas > 95% had IgG to triaose- and pentaose Forssman antigens.

Even within the relatively similar classes of antigens, there are discrete differences observable. Many individuals have antibodies against 4 of the 5 chitin glycans (tri- to heptasaccharide), to 5 of the 7 Forssman-related glycans, and to 4 of the 5 Globo antigens. The top 10 most common glycan antigens, bound by both anti-carbohydrate IgG and IgM are shown in Supplementary Fig. S1, and represent a unique finding in our study.

Strikingly, the highest binding signal for both IgG and IgM ACAs was the three Forssman antigens, isoForssman antigen pentaose (#49) GalNAcα1-3GalNAcβ1-3Galα1-3Galβ1-4Glc-AEAB, Forssman antigen pentaose (#45) GalNAcα1-3GalNAcβ1-3Galα1-4Galβ1-4Glc-AEAB and Forssman antigen triaose (#48) GalNAcα1-3GalNAcβ1-3Gal-AEAB. We also observed more anti-carbohydrate IgG responses to longer chitin chains (GlcNAcβ1-4GlcNAc)_n_ yet less binding to longer β-glucan chains (Glcβ1-4Glc)_n_. We did not observe this trend for IgMs against chitins. Donors either have IgM antibodies that bind to chitins of all lengths, or none at all. People with high IgG levels toward chitin antigens have relatively high levels of IgM toward the same oligosaccharides, while individuals with strong IgM signals toward the chitin antigens have only a weak respective IgG signal. Donors 53, 74 and 93 are good examples for this distribution of IgG and IgM antibodies toward chitins.

It might be expected that ACAs toward ganglioside antigens would be relatively rare, as antibodies to self-gangliosides are associated with autoimmune diseases and autoimmune peripheral neuropathies^62^. There were, however, several individuals with both IgG and IgM ACAs to certain gangliosides. Interestingly, only three individuals (donors 9, 15 and 80) show IgG antibodies toward multiple ganglioside glycans. The ganglioside GM1b (glycan #39) was the most commonly recognized ganglioside by anti-carbohydrate IgG and IgM throughout the population. *Campylobacter jejuni* expresses this ganglioside structure on its surface in the core of its LPS; such an infection, which is common and associated with gastroenteritis worldwide^63^, might lead to such antibodies toward GM1b^64^. IgG antibodies to the minor ganglioside GM1b are also frequently present in sera of patients with Guillain-Barré syndrome^29–31^. Interestingly, we observed no individuals that had IgG or IgM antibodies to the disialylated ganglioside GD2. This ganglioside is highly expressed in many human tumors^65^, and shows promise as a vaccine for treating melanoma^66^.

Many individuals expressed IgM toward HNK1 related antigens, Forssman, and Globo series antigens. The HNK1 epitope, glycan #70 HSO_3_-3GlcAβ1-3Galβ1-4Glc-AEAB and #71 HSO_3_-3GlcAβ1-3Galβ1-3GlcNAcβ1-3Galβ1-4Glc-AEAB were bound by IgM, but not bound by IgG. One individual had IgG but not IgM to Sialyl Lewis x (glycan #51 and #52), and 11 donors had IgM to either glycan #72 or #73 (isoglobotetraose and isoglobopentaose glycans).

In contrast to conventional predictions that people do not generally make such antibodies to the H epitope Fucα1-2Galβ1-R, we found the anti-O(H) repertoire to be unusual. The most common O(H) antigen recognized by IgG antibodies in 37% of the individuals is glycan #81, isoglobo-H analogue type 1 Fucα1-2Galβ1-3GalNAcβ1-3Galα1-3Galβ1-4Glc-AEAB. Humans presumably cannot synthesize this glycan, as the iGb3 gene in humans is inactive, yet active in pigs^67^. In 28% of all individuals, glycan #82, Globo-H hexaose Fucα1-2Galβ1-3GalNAcβ1-3Galα1-4Galβ1-4Glc-AEAB was found, with some individuals showing strong IgM antibody binding. Globo-H has been found to be rarely expressed in normal human tissue and is thought of as a human tumor-associated carbohydrate antigen^68^. In many individuals, binding of IgG ACAs was more skewed toward anti-blood type A and B antigens (Fig. 2A), whereas IgM ACAs generally bound all blood type A, B, and O(H) antigens equivalently (Fig. 2B). Interestingly, the bar plots that represent the total IgM antibody signal per glycan of the whole population shows only minor differences between the three blood group antigen families.

### Antibodies recognizing specific oligosaccharides do not cross-react with other glycans

**Figure 7:**
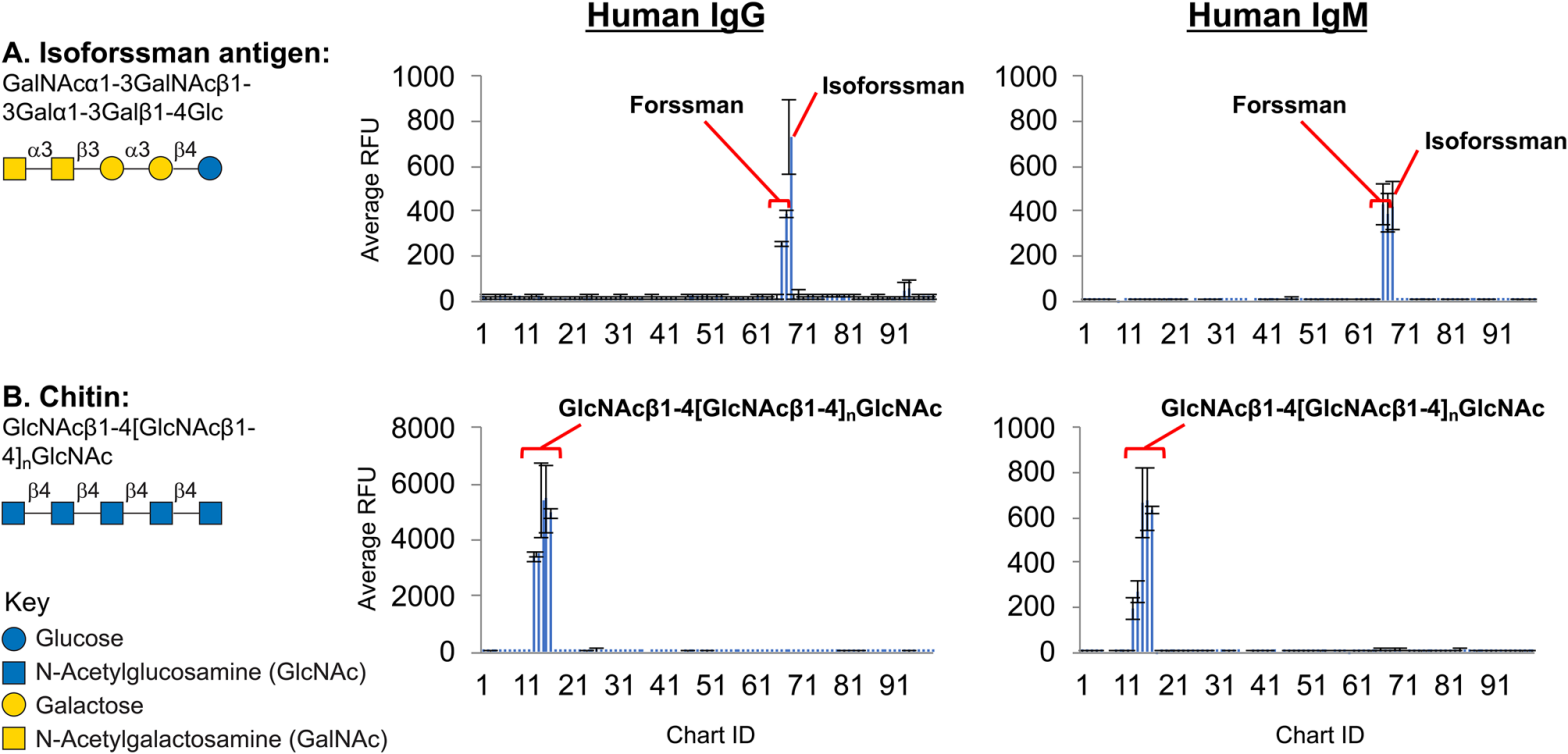
Antibodies (IgG and IgM) were isolated from human serum using glycan magnetic beads coupled to isoForssman (**A**) and chitin (**B**), and screened for their specificity on the NCFGv1 microarray.

To test the specificity of the anti-carbohydrate antibodies detected on glycan microarrays, we chose several glycan antigens with high recognition by anti-carbohydrate IgG and IgM in many of the donors, and directly affinity purified the antibodies from each sera. Using a targeted approach with glycan-coated magnetic beads, we pulled down IgG and IgM antibodies and subsequently tested their specificity on the NCFGv1 microarray. We used chitin-coated magnetic beads and NHS functionalized magnetic beads coupled with the AEAB-derivatized isoForssman antigen GalNAcα1-3GalNAcβ1-3Galα1-3Galβ1-4Glc-AEAB to successfully enrich highly specific antibodies; in subsequent retesting on the glycan microarray these purified antibodies showed little to no cross-reactivity with any other tested antigens lacking these glycan epitopes (Fig. 7). These results demonstrate that individual antibody responses to distinct antigens generally have a high degree of specificity for that particular antigenic epitope or determinant with no appreciable cross-reactivity.

### Immunological aging and ethnicity

**Figure 8:**
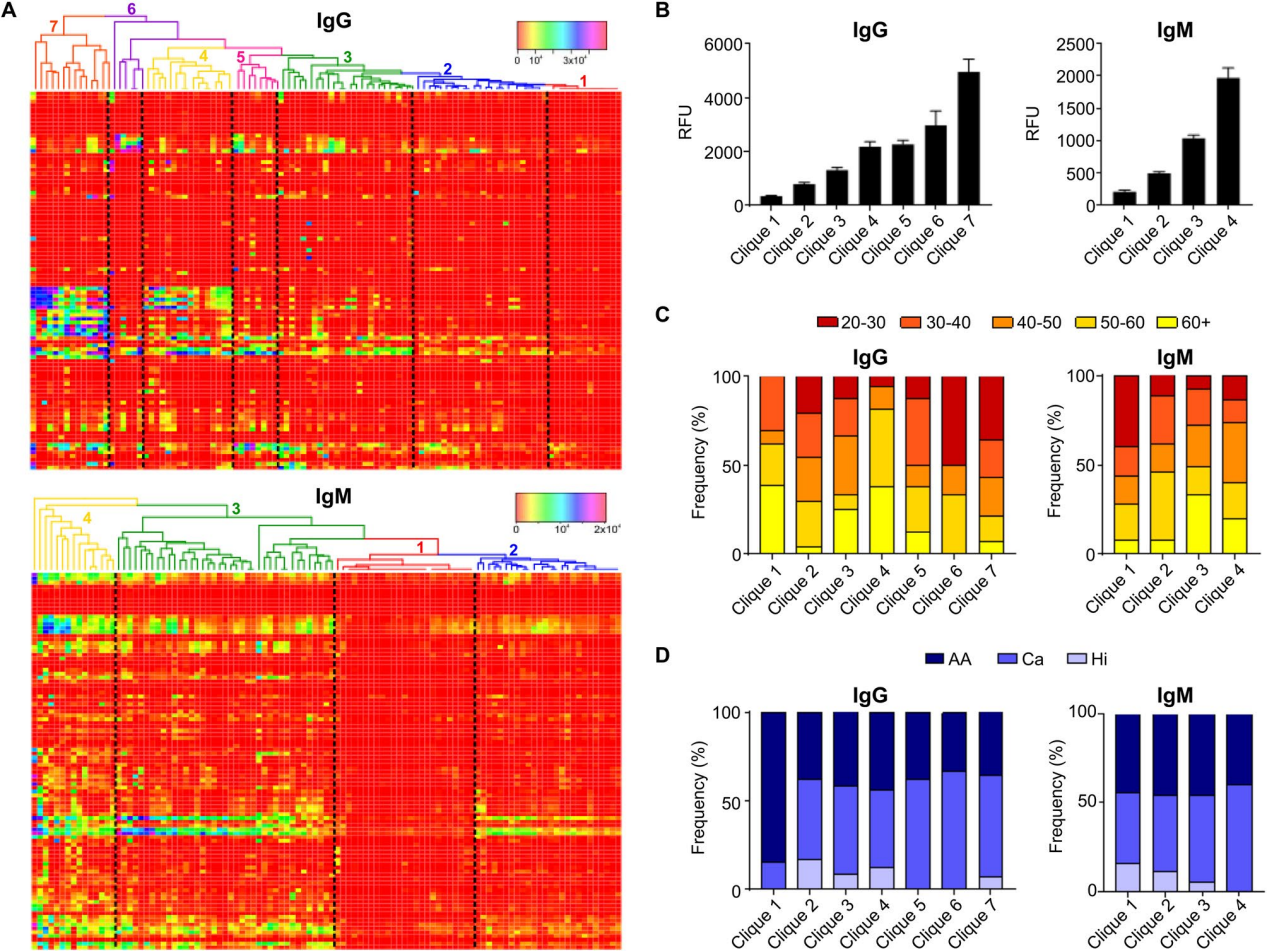
(**A**) Cliques of individual IgG (top) or IgM (bottom) immunoprofiles with correlated anti-carbohydrate reactivities computed by the dendrogram clustering algorithm. Clique-associated color-coded lines of dendrogram tree, color key and distribution histogram are depicted. (**B**) Averaged RFU levels of cliques segregated by anti-glycan reactivity. (**C**,**D**) Clique distribution of IgG or IgM immunoprofiles related to age (**C**) or ethnicity (**D**). *AA* African-American; *Ca* Caucasian; *Hi* Hispanic.

To further understand the individual IgG or IgM ACAR immunoprofiles, we employed hierarchical clustering analysis^14^. Seven major subgroups with highly correlated/clustered glycan reactivities were identified for IgG, and four subgroups for IgM (Fig. 8A), which we further categorized based on their averaged binding intensities from low (clique 1) to high (IgG: clique 7; IgM: clique 4) (Fig. 8B). Frequency distribution analysis revealed an ACAR association with age, as individuals below the age of 30 were over represented in IgG high (cliques 6 and 7) and IgM low (clique 1) reactivity subgroups (Fig. 8C). Individuals in the age range of 20–29 years were under represented in IgM cliques 2–4 (7.7–13.3%). In contrast, individuals above the age of 40 clustered in subgroups with only moderate (clique 4: 93.8%) to low (cliques 1–3: 54.2–69.2%) IgG, but dominant IgM (cliques 3 and 4: 71.2–73.3%) anti-carbohydrate reactivities. In regard to ethnicity, in IgG clique 1, with the lowest IgG anti-carbohydrate reactivities, as much as 84.6% among the ACAR immunoprofiles were African-American (AA) individuals (n = 11) (Fig. 8D), which stands for the 23.4% of the total AA individuals (n = 47) included in the study. This subset was heterogenous in gender and age, but older than 30 years. Otherwise, no remarkable differences among cliques were found in terms of ethnicity or gender (Supplementary Fig. S2). Taken together, these data indicate an IgG-predominant ACAR in younger age groups and point towards a loss of class-switching capabilities in the context of immunological aging. Furthermore, a limited capacity to generate anti-carbohydrate antibodies of the IgG subclass was observed in a subset of African-Americans above 30 years of age.

## Discussion

Our results indicate that the sera of each individual tested contains a relatively unique ACAR comprised of IgG and IgM classes and includes antibodies recognizing an extensive variety of carbohydrate antigens. Antibodies to microbial glycans on the microbial glycan microarray indicate that some of the carbohydrate antigens are related to those in microbes, whereas other antibodies are to animal-related glycans of both human and non-human origin. Our results reveal a rich variety of glycan antigens recognized by human antibodies, and show that the IgG versus IgM repertoires in an individual lack strong correlation. The differences in individual ACARs suggest a wide variety of exposures to carbohydrate antigens in the environment, as might be expected with varied individual backgrounds; it also suggests potential individual differences in the ability to generate antibodies. The results lead us to hypothesize that these types of analyses defining the ACAR of an individual might provide insight into the health, disease exposure, and susceptibility of an individual.

This current study was undertaken following our recent observations that individuals identified to have a primary antibody or immunodeficiency disorder lack IgG to carbohydrate antigens and some are specifically unable to mount protective immunity to carbohydrate-based vaccines^25^. These observations on antibody disorders were themselves a follow-up of our earlier observations noting the extensive assortment of anti-carbohydrate antibodies in IVIG^13,14,28^, commercial sources of pooled IgG from thousands of individuals. We found that although many of the different commercial sources of IVIG shared some anti-carbohydrate antibody profiles, there were significant differences, suggesting that the sources of IgG for commercial preparations, while pooled from many individual sera, might still represent differences in donor populations^13,14^. Furthermore, these studies only examined IgG, not IgM.

It is commonly believed that the major immune responses to carbohydrate antigens are typically biased toward IgM, because it has been assumed that glycans are not highly antigenic. Obviously, this is not the case in natural settings; glycoconjugates in which sugars are linked to proteins or lipids are highly immunogenic and IgG can be directly generated to glycan antigens in that context in rabbits, mice, and humans^18^. In addition, prior studies using glycan microarray technology also supported the possibility that human sera contain a wide range of anti-carbohydrate antibodies of IgG nature^11,17,19,21,50–52,69^.

Although prior studies have not well explored the relative contributions of IgG versus IgM toward specific carbohydrate antigens, when IgG and IgM were comparatively tested, the results suggested some relationships between IgG and IgM repertoires to glycans^69^, but in some cases the samples sizes were relatively small, thus limiting the conclusions. Our results demonstrate that while there appear to be stronger relationships of IgM and IgG ACARs within an individual as compared to different individuals, nevertheless, there are wide variations. We do not have any information yet as to the reasons for the lack of correlation between IgM and IgG anti-carbohydrate repertoires in an individual. Some individuals exhibit polysaccharide antibody production deficiency and lack the ability to generate IgG anti-carbohydrate antibodies to certain antigens, but perhaps are not deficient in producing other antibody isotypes^70^, further raising questions about the origins of anti-carbohydrate antibodies, class-switch and antibody persistence. We observed an IgG-predominant ACAR in younger age groups and a more limited capacity to generate anti-carbohydrate antibodies of the IgG subclass in a subset of African-Americans above 30 years of age. These studies are consistent with others that noted a contribution of age on the anti-carbohydrate responses^17^, but more studies are needed with larger cohorts to draw strong conclusions, and with more detailed information about the clinical and health history of donors.

A prior study examined a number of normal samples for antibodies to glycans^22^, and observed binding to several dozen of the present glycan motifs we have also identified. That study did not analyze samples, however, for recognition of chitin, Forssman, and HNK antigens; neither were samples grouped according to ethnicity or age of donors, nor were the differences in binding of IgG and IgM parsed out^22^. But similar to our study, that prior study also noted the relatively low level of antibodies to the α-Gal epitope. Overall, our results are consistent with the results of others and indicate that vast numbers of ACAs are present in the human serum. Interestingly, while in our study we were unable to assess whether the ACARs in this large set of individuals is stable over time, recent studies using similar approaches, but a smaller set of individual sera, indicate that the expression of anti-carbohydrate antibodies are relatively stable over weeks to months^69^.

Prior studies have suggested that the antibodies to blood group antigens and α-Gal-type antigens are present at high-titer in human sera^15,20,71^, but that is not what we observed in our comparative study. Instead, the highest reactivities and most commonly observed antibodies were toward chitin oligosaccharides, Forssman antigens, and globoside (see the top 10 antigenic carbohydrates in Supplementary Fig. S1). Interestingly, many of the prior studies on anti-carbohydrate antibodies in serum did not report antibodies to chitin or globoside-related oligosaccharides^17,72^, or to Forssman oligosaccharides beyond the simple disaccharide^17,72^. The prevalence of antibodies to these antigens suggest widespread and common exposure to such antigens in the environment.

Because we observed so many antigens to be recognized by ACAs, it is not practical to discuss each one individually here. But in regard to a few of the observed antigens, some important observations can be made. The Forssman antigen was discovered in 1911 as an antigen in guinea pig and sheep erythrocytes^73^, but not rabbits. Early studies suggested that antibody to Forssman in human sera was primarily IgM^74^, and that anti-Forssman antibodies in people might be common^75^. The Forssman non-reducing terminal disaccharide sequence GalNAcα1-3GalNAc is expressed by Group C Streptococcus^76^ where it is linked to rhamnose^77^, and may be responsible for provoking the antibodies to the Forssman glycolipid. Interestingly, we saw no correlation between responses to the terminal A blood group glycans, versus those to Forssman antigens. Both A blood group and Forssman antigens have terminal GalNAcα1–3R, but in the former the underlying structure is GalNAcα1–3(Fucα1-2)Galβ1-R, whereas in the latter it is GalNAcα1–3GalNAcβ1–3R. This indicates an exquisite recognition by antibodies, far beyond the potentially important terminal sugar and its linkage. Also of interest is the recent finding that some people express the Forssman antigen due to a reversion in the Forssman synthetase gene^78^, which all humans normally inherit as an inactive allele. Such Forssman-positive individuals lack anti-Forssman antibodies. There are prior studies demonstrating that vaccination with the disaccharide GalNAcα1–3GalNAcβ, which is only a portion of the Forssman antigen, results in antibodies to such an antigen and that those responses are associated with survival in patients with prostate cancer^57^.

We observed antibodies to chitin in most individuals, and it should be noted that chitin is commonly made by all fungi, as well as all insects, arthropods, and crustaceans. There is evidence that chitin and its glycans may be important in understanding some human diseases, as was shown for Crohn’s disease, where fungal chitin may be proinflammatory^79^. It has also been shown that *Candida albicans* infection can induce anti-chitin antibodies; thus, these antibodies have been proposed as a potential biomarker for Crohn’s disease^80^. These and many other such studies on immunogenic glycans to which we are exposed clearly show the need to understand the generation of an individual’s ACAR and its potential relationship and utility in disease diagnostics and pathology.

An obvious outcome of our results is the consideration that each individual has a type of “ACAR-barcode” in which the ACAR readout is relatively unique. As to the origin of such individual ACARs, we can only speculate that it is related to an individual’s exposure to environmental antigens, e.g. derived from insects, fungi, bacteria, plants, animals, etc. If so, and this remains to be firmly established, it would indicate that the ACAR of an individual represents a partial immunological record of exposure of each individual to carbohydrate antigens. We have observed this previously, in fact, for individuals infected with various pathogens, including parasitic worms such as *Schistosoma mansoni* and others^35,53,81–93^, as well as in studies on children infected with the protozoan *Cryptosporidium parvum*^34^, all of whom indicate that specific anti-carbohydrate antibodies are induced only in those individuals infected by specific pathogens. Such studies are also consistent with many historical studies showing that bacterial exposure can lead to specific antibody repertoires in animals^94,95^.

There are several limitations to our studies to note. We chose a specific dilution of sera to analyze based on prior studies and thus, we did not closely examine the titers of antibodies to individual glycans, nor the specific subclasses of IgG and levels of each recognizing each glycan. Such parameters might be examined in future studies depending on the availability of substantial amounts of glycans for such larger-scale analyses. Many prior studies have reported that antibodies to polysaccharides are most commonly of the IgG2 subclass^96,97^ and that lacking IgG2 may lead to less protection against all pneumococcal serotypes^98^, but in our past studies we observed that other subclasses of IgG also recognize glycan antigens^13^. Additional studies that parse out the subclasses of IgG towards these glycans and how they may relate to health status and exposure will be an important component of individualized medicine. We also did not examine IgA or IgE antibodies to carbohydrates, but recent studies indicate that each individual probably also possesses some degree of unique IgA antibodies to glycan antigens, albeit at lower levels of antibody, when explored using glycan microarray formats^69,99^. Finally, we have not examined the ACARs of individuals in terms of disease exposure, history, health, or other individual parameters that could be informative, in addition to longitudinal studies over extended periods of time. These are clearly measurements to conduct in future studies, based on our findings here.

Our study has many important implications. The results indicate that each individual has a type of ACAR-barcode which could be identified quickly and easily using the approaches herein, and these snapshots of immune status could provide information as to disease relationships in the individual. The early diagnosis of primary antibody or immunodeficiency disorders could also be potentially aided by examining the ACAR of those suspected to have deficiencies in producing anti-carbohydrate IgG^25^. Potentially, the ACAR-barcode could be examined in terms of the genome and glycoproteome/glycome of an individual to identify any genetic relationships that may contribute to the development of the ACAR-barcode. Finally, in terms of personalized and predictive medicine, the ACAR-barcode is easy to identify by the methods described here, using a minimal amount of serum, and will determine the presence of antibodies, but just as importantly identify the lack of antibodies to specific glycans that are being tested. This might provide useful information and potential biomarkers as an adjunct to other ‘omics’ insights currently needed for example, for the formulation of vaccines^100^, developing new cancer biomarkers^101^ and precision oncology^102^, understanding the disparities in drug efficacy, relationships of age, gender, and ethnicity to disease, and understanding cardiovascular disease and heart failure^103^. In regard to the latter, it should be noted that anti-carbohydrate antibodies generated by infection with group A *Streptococcus* are associated with acute rheumatic fever, which can lead to damage to cardiac valves expressing recognized carbohydrate antigens^104^. With the knowledge that antibodies to carbohydrate antigens are associated with neurodegenerative disorders such as Guillain-Barré Syndrome^29–31^, the relationship of the ACAR of an individual to such disorders could also be important to study. The ACAR-barcode could be a useful and unique identifier in disease exposure, susceptibility, and diagnosis.

## Supplementary information

Supplementary file1

Supplementary file2

## Data Availability

The processed NCFGv1 glycan microarray data acquired for this paper are provided in Supplementary as an excel file (unprocessed data available upon request). In addition, we have uploaded a GLAD session file for analysis using GLAD (GLycan Array Dashboard^105^) and provide the data files for the CFG and MGM glycan microarrays as downloadable files here: https://ncfg.hms.harvard.edu/ncfg-data/microarray-data/healthy-human-serum-anti-carbohydrate-antibodies
