# Supplementary material for: Unique repertoire of anti-carbohydrate antibodies in individual human serum
Source: Sci Rep. 2020 Sep 22;10:15436. doi: 10.1038/s41598-020-71967-y (PMC7509809; doi:10.1038/s41598-020-71967-y)

**Supplementary Information**

**Unique Repertoire of Anti-Carbohydrate Antibodies in**

**Individual Human Serum**

Ralph N.D. Luetscher^1,2*^, Tanya R. McKitrick^1*^, Chao Gao^1^, Akul Y. Mehta^1^,

Alyssa M. McQuillan^1^, Robert Kardish^1,3^, Kayluz Frias Boligan^4^, Xuezheng Song^5^, Lenette Lu^6,7^, Jamie Heimburg-Molinaro^1^, Stephan von Gunten^4^, Galit Alter^6^, and Richard D. Cummings^1,8^

**Supplementary Information Legends**

**Supplementary Table S1.** The donor ID#, age, gender and ethnicity of the healthy individual donors screened on the NCFGv1 glycan microarrays. N = the number of individuals in each category.

**Supplementary Table S2**. Common names and structures of the glycans printed on the NCFGv1 microarray. (*Excel file- tab 1*)

**Supplementary Table S3.** Six pairs of individuals had nearly identical binding profiles for IgG and IgM, as shown by the Pearson coefficient, however this comparison was relative rare among the individuals tested.

**Supplementary Figure S1**. The name and structure of the top 10 carbohydrate antigens bound by both anti-carbohydrate IgG and IgM in human serum.

**Supplementary Figure S2.** Gender distribution in IgG or IgM immunoprofile computed by the dendrogram clustering algorithm.

**Supplementary Data File**- Processed NCFGv1 microarray data for IgG and IgM for all 105 donors, shown as the average relative fluorescent units (RFU) from the four printed glycan replicates, along with the standard deviation (SD) and the coefficient of variance (%CV) (*Excel file- tabs 2-3*). Raw, unprocessed data available upon request.

**Supplementary Table S1**

| **ID#** | **Age** | **Gender** | **Ethnicity** | **N** |
| --- | --- | --- | --- | --- |
| 1-5 | 20-29 | Female | Caucasian | 5 |
| 6-9 | 20-29 | Female | African-American | 4 |
| 10-14 | 20-29 | Male | Caucasian | 5 |
| 15-17 | 20-29 | Male | African-American | 3 |
| 18 | 20-29 | Male | Hispanic | 1 |
| 19-23 | 30-39 | Female | Caucasian | 5 |
| 24-28 | 30-39 | Female | African-American | 5 |
| 29-33 | 30-39 | Male | Caucasian | 5 |
| 34-38 | 30-39 | Male | African-American | 5 |
| 39 | 30-39 | Male | Hispanic | 1 |
| 40-44 | 40-49 | Female | Caucasian | 5 |
| 45-49 | 40-49 | Female | African-American | 5 |
| 50-53 | 40-49 | Male | Caucasian | 4 |
| 54-58 | 40-49 | Male | African-American | 5 |
| 59-61 | 40-49 | Male | Hispanic | 3 |
| 62-66 | 50-59 | Female | Caucasian | 5 |
| 67-71 | 50-59 | Female | African-American | 5 |
| 72-76 | 50-59 | Male | Caucasian | 5 |
| 77-81 | 50-59 | Male | African-American | 5 |
| 82-85 | 50-59 | Male | Hispanic | 4 |
| 86-89 | 60+ | Female | Caucasian | 5 |
| 91-95 | 60+ | Female | African-American | 5 |
| 96-100 | 60+ | Male | Caucasian | 5 |
| 101-105 | 60+ | Male | African-American | 5 |
|  |  |  | Total | 105 |

**Supplementary Table S3**

|  |  | **Pearson coefficient (*r*)** | |
| --- | --- | --- | --- |
| **Individual Donor A** | **Individual Donor B** | **IgG** | **IgM** |
| #3 (F-Ca-20) | #19 (F-Ca-20) | 0.985 | 0.912 |
| #12 (M-Ca-20) | #18 (M-Hi-20) | 0.986 | 0.932 |
| #26 (F-AA-30) | #46 (F-AA-40) | 0.984 | 0.958 |
| #50 (M-Ca-40) | #72 (M-Ca-50) | 0.995 | 0.946 |
| #75 (M-Ca-50) | #97 (M-Ca-60) | 0.985 | 0.985 |
| #78 (M-AA-50) | #102 (M-AA-60) | 0.989 | 0.982 |

**Supplementary Figure S1**


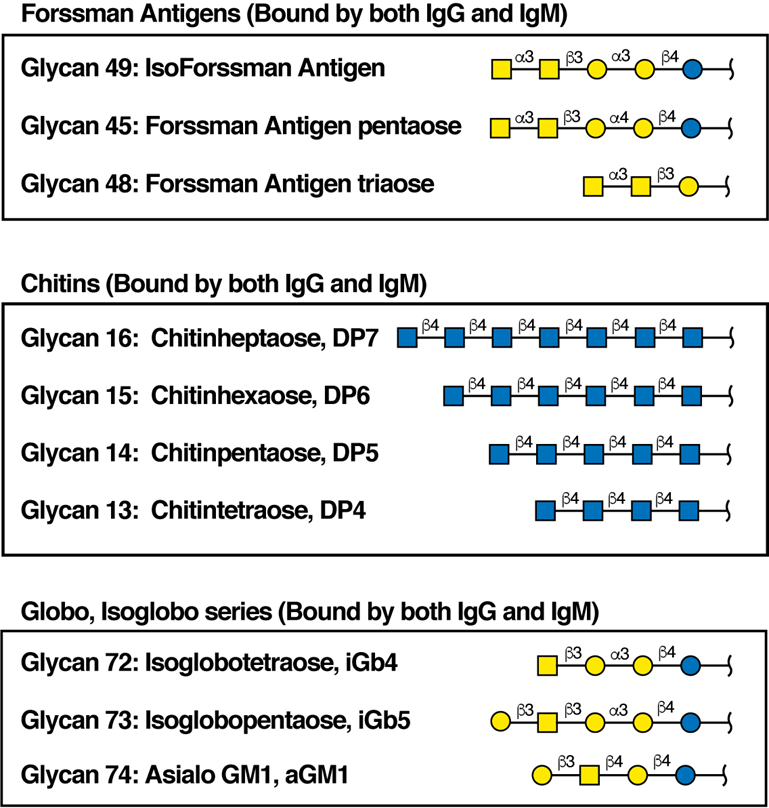


**Supplementary Figure S2**


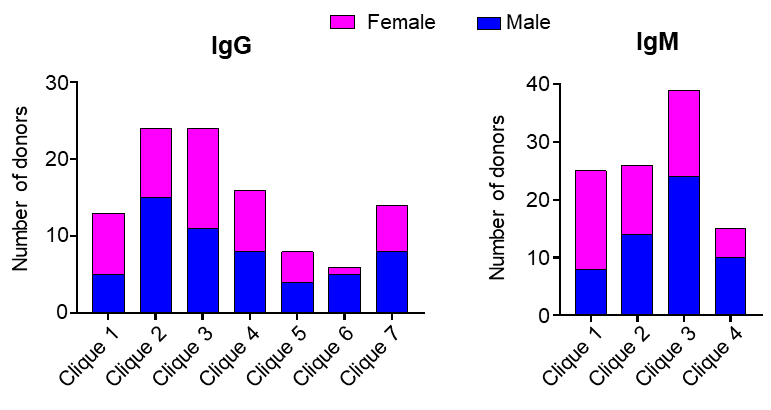

Supplement: Supplementary file 1 — Supplementary file1 [file 41598_2020_71967_MOESM1_ESM.docx]
